# Oogenesis in cultures derived from adult human ovaries

**DOI:** 10.1186/1477-7827-3-17

**Published:** 2005-05-05

**Authors:** Antonin Bukovsky, Marta Svetlikova, Michael R Caudle

**Affiliations:** 1 Laboratory of Development, Differentiation and Cancer, Department of Obstetrics and Gynecology, The University of Tennessee Graduate School of Medicine, Knoxville, Tennessee, USA

## Abstract

Ten years ago, we reported that in adult human females the ovarian surface epithelium (OSE) is a source of germ cells. Recently, we also demonstrated that new primary follicles are formed by assembly of oocytes with nests of primitive granulosa cells in the ovarian cortex. The components of the new primary follicles, primitive granulosa and germ cells, differentiated sequentially from the OSE, which arises from cytokeratin positive mesenchymal progenitor cells residing in the ovarian tunica albuginea. In the present study, we investigated the possibility that the oocytes and granulosa cells may differentiate in cultures derived from adult human ovaries. Cells were scrapped from the surface of ovaries and cultured for 5 to 6 days, in the presence or absence of estrogenic stimuli [phenol red (PhR)]. The OSE cells cultured in the medium without PhR differentiated into small (15 micron) cells of granulosa phenotype, and epithelial, neural, and mesenchymal type cells. In contrast, OSE cells cultured in the presence of PhR differentiated directly into large (180 micron) cells of the oocyte phenotype. Such cells exhibited germinal vesicle breakdown, expulsion of the polar body, and surface expression of zona pellucida proteins, i.e. characteristics of secondary oocytes. These *in vitro *studies confirm our *in vivo *observations that in adult human ovaries, the OSE is a bipotent source of oocytes and granulosa cells. Development of numerous mature oocytes from adult ovarian stem cells *in vitro *offers new strategies for the egg preservation, IVF utilization, and treatment of female infertility. In addition, other clinical applications aiming to utilize stem cells, and basic stem cell research as well, may employ totipotent embryonic stem cells developing from fertilized oocytes.

## Background

The origin of oocytes (and primary follicles) in ovaries of adult mammalian females has been a matter of dispute for over one hundred years. The resulting dogma was that all oocytes in adult mammalian females persist from the fetal period of life [1]. However, from a phylogenetic viewpoint, it seems contradictory that mammalian females, including humans, would evolve a uniquely retrogressive reproductive mechanism, requiring preservation of their gametes from the fetal period for up to several decades. Such long lasting preservation could cause an accumulation of spontaneous or environmentally induced genetic alterations of oocytes in resting primary follicles. On the contrary, oogenesis has been demonstrated in cultured mouse embryonic stem cells [2], and mitotically active germ cells have been reported in ovaries of adult prosimian primates [3] and mice [4]. We have shown that mesenchymal cells in ovarian tunica albuginea (TA) differentiate into surface epithelium, a source of germ cells entering blood vessels and contributing to follicular renewal in adult human females [5,6]. These reports represent challenges to established dogma on the fetal origin of mammalian eggs [7,8].

Regarding follicular renewal in adult human females, our reports provide direct evidence that the ovarian surface epithelium (OSE) is a source of germ cells, and new primary follicles are formed by assembly of oocytes with nests of primitive granulosa cells in the ovarian cortex [5,6]. Components for the new primary follicles, primitive granulosa and germ cells, differentiate sequentially and *de novo *from mesenchymal progenitor cells residing in the ovarian TA. It appears that mesenchymal progenitor cells first contribute to the development of epithelial cells similar to granulosa cells, and these cells subsequently form epithelial nests descending into the deeper ovarian cortex. Oogenesis follows later. During this period, the mesenchymal progenitor cells differentiate into OSE cells with an embryonic character, lining either the ovarian surface or invaginated epithelial crypts. These cells are a source of germ cells, which assemble with nests of primitive granulosa cells to form primary follicles. Systemic hormonal concentrations may influence these processes. The data presented here indicate that granulosa cells are likely to develop during a period of low estrogenic stimuli (early and mid follicular phase). Differentiation of germ cells and oocytes may be associated with high estrogen concentrations (preovulatory period). The assembly of oocytes with granulosa cell nests (follicular renewal) may occur during the luteal phase [6]. Differentiation of primitive granulosa and germ cells from the bipotent mesenchymal cell precursors of TA in adult human ovaries possibly represents a sophisticated mechanism created during the evolution of female reproduction. This contrasts with the continuous preservation of germline stem cells seen in males [9], and female prosimian primates and mice [3,4].

In culture, the OSE cells undergo an epithelio-mesenchymal transition. These cells are initially cytokeratin (CK) positive, but lose CK expression with time and passage in culture [10]. However, upon introduction of E-cadherin, the mesenchymal type cells can differentiate backward into the epithelial phenotype [11]. Multipotential progenitor cells exhibiting a mesenchymal phenotype and capable of differentiation into distinct cell types have been derived from various other adult tissues [12-16]. Mesenchymal-epithelial and epithelial-mesenchymal transitions may reflect a plasticity of progenitor cells in a particular microenvironment. They may occur sequentially (mesenchymal-epithelial followed by epithelial-mesenchymal transitions) under the influence of the extracellular matrix, cytokines (transforming growth factor β, fibroblast growth factor, hepatocyte growth factor, epidermal growth factor, BMP2 and BMP4), adhesion molecules (integrins, E-cadherins), membrane receptors, intercellular junctions, signaling pathways (MAPK) or transcription factors (β-catenin), commonly produced in the embryo and less frequently in adult organisms. Such transitions are manifestations of cell plasticity and result in dramatic changes – in lineage commitments to certain cell types (reviewed in [17]).

In culture, in addition to their characteristic morphology, oocytes can also be identified by alkaline phosphatase activity, though nonspecific results have been described in various tissues [18]. However, zona pellucida (ZP) proteins and some ZP antigens, such as PS1 meiotically expressed carbohydrate antigen and heat-solubilized porcine zona (HSPZ) proteins, are more specific markers. In fact, they have been detected in the OSE cells of the rabbit, cat, monkey, baboon and human [6,19,20]. Hence, expression of ZP proteins in OSE cells suggests a relationship to oocytes. The oocytes may also express CK18, a marker of the Balbiani body, but not CK5,6,8,17 markers of OSE and granulosa cells (reviewed in [6]). Furthermore, oocytes also express the intermediate filament vimentin, a protein which plays an important role in the maturation and fertilization of eggs [21].

Our goal for the present study was to investigate mesenchymal-epithelial and epithelial-mesenchymal transitions in OSE cell cultures from adult human ovaries. An *in vitro *approach allows control of the conditions in the initial medium composition, e.g., presence or absence of estrogenic stimuli, and determination of the secretory products of cultured cells in the conditioned medium. The study of OSE cultures was initially a part of a larger project to establish primary fibroblast cultures from the pelvic floor (levator ani fascia) and uterine round ligaments. Our preliminary studies indicated that pelvic floor fibroblasts grow better in the presence of estrogenic stimuli. We also detected the presence of oocyte phenotype cells in some OSE cultures. Thus, in the present experiments, we cultured OSE cells in the presence or absence of estrogenic stimuli. Phase contract microscopy and antibodies against HSPZ, PS1, CK18, CK5,6,8,17, and vimentin were used in single and double color peroxidase immunohistochemistry to identify oocytes and granulosa cells in cultures derived from adult human ovaries.

## Materials and methods

### Collection and cultures of OSE cells

All chemicals and consumables, except where specified otherwise, were purchased from Sigma Chemical Co., St. Louis, MO. Cultured cells were collected from ovaries associated with fresh hysterectomy/bilateral salpingo-oophorectomy specimens (five women, ages 39 – 52 years). The surgery was performed for medical indications, including chronic pelvic pain, uterine fibroids, and/or uterine bleeding (severe dysmenorrhea) not responding to the conservative treatment. The study was approved by the Institutional Review Board and informed consent was obtained from the patients.

**Figure 1:**
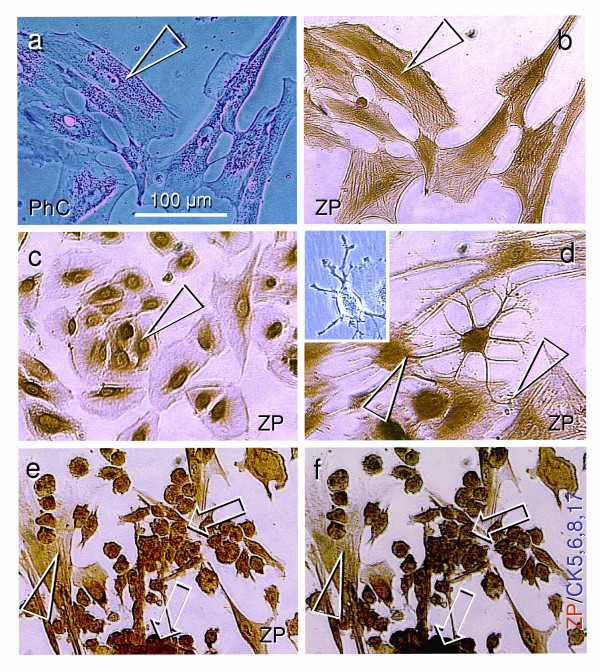
**Cell phenotypes in 6-day OSE culture maintained in phenol red free DMEM/F12 medium. a**) Live fibroblast type cells in phase contrast microscopy (PhC) show perinuclear accumulation of organelles (arrowhead). **b**), Immunostaining of the same cells with HSPZ antibody shows ZP+ nuclei and perinuclear organelles (arrowhead). **c**) A cluster of cells showing epithelial phenotype with ZP+ nuclei and perinuclear organelles (arrowhead). **d**) ZP+ neural type cell shows extensions toward the ZP+ perinuclear staining (arrowhead) of neighboring mesenchymal type cells. Inset shows similar type of cell in the live culture. **e**) Clusters (arrows) of small (15 μm) cells of granulosa cell phenotype with strong ZP expression. Arrowhead indicates mesenchymal type cells. **f**) Double staining for ZP/CK5,6,8,17 (blue substrate) shows dark-blue staining of small cell clusters (arrows), but mesenchymal type cells remain brown only (arrowhead).

**Figure 2:**
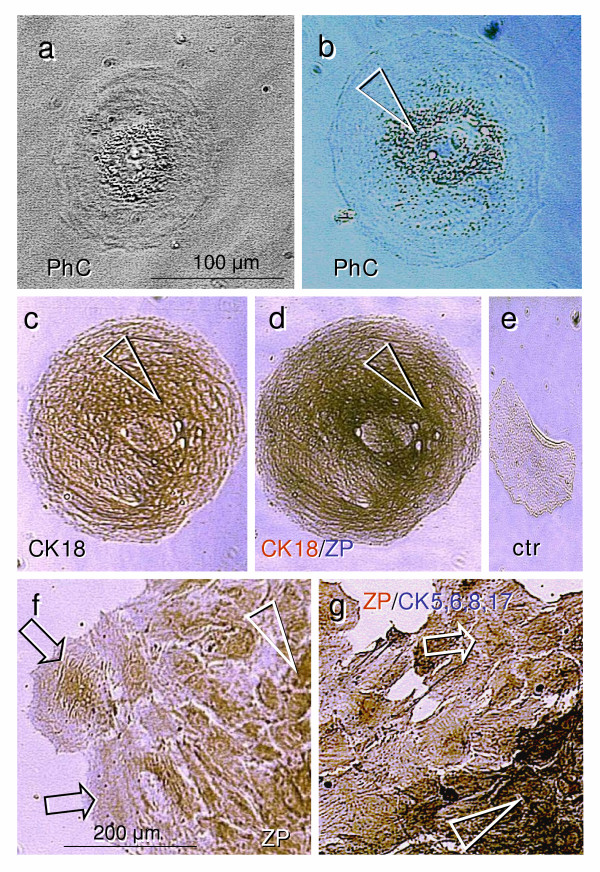
**Five-day OSE culture maintained in DMEM-HG medium with phenol red**. **a**) Phase contrast microscopy of a live cell with a large oocyte phenotype (120 μm in diameter) shows a centrally located nucleus with nucleolus and perinuclear accumulation of cell organelles. **b**) Larger cell reaching 180 μm shows similar morphology. **c**) The cell presented in panel **b **stained for CK18 shows cytoplasmic staining with an accumulation of CK18 immunoexpression in the perinuclear space (arrowhead). **d**) The same cell in CK18 (brown) and ZP (blue) double color immunostaining (CK/ZP) shows high density of ZP expression in the perinuclear space (compare with panel **c**). **e**) Control staining shows no reactions in OSE cells when the anti-HLA DR antibody was used in place of the primary antibody. **f**) A cluster of cells exhibiting epithelial phenotype shows nuclear ZP expression (arrowhead) and differentiation into large cells (arrows). **g**) Another cluster subjected to the ZP/CK,5,6,8,17 double staining shows dark-blue staining of smaller epithelial cells (arrowhead), which diminishes in more differentiated larger cells (arrow). Bar in (**a**) for **a-e**.

**Figure 3:**
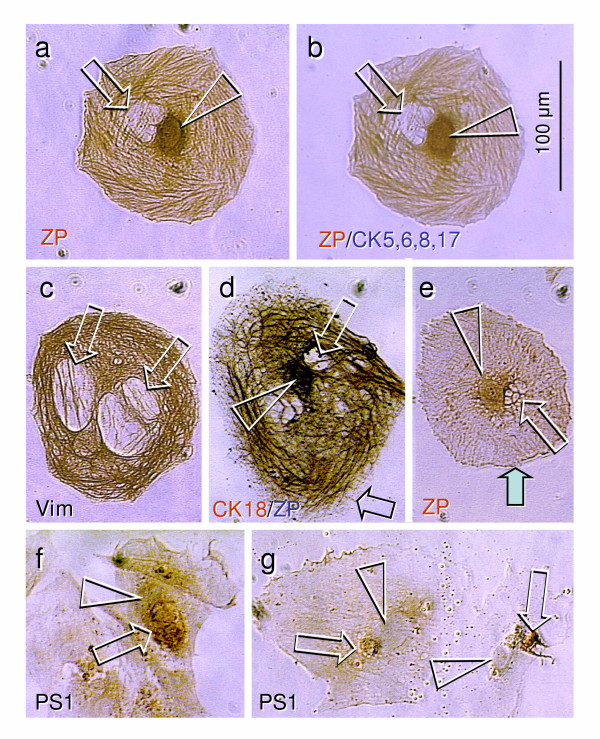
**Five-day OSE culture maintained in the DMEM-HG medium with phenol red as in Fig. 2. a**) Cell of oocyte phenotype with two nuclei. The centrally located nucleus shows ZP immunostaining (arrowhead) but adjacent nucleus is unstained (arrow). Note ZP+ intermediate filaments. **b**) The same cell subjected to double color (ZP/CK5,6,8,17) immunohistochemistry shows no additional (dark blue) staining. **c**) Another large cell stained for vimentin shows expression in intermediate filaments and two unstained large nuclei (arrows). **d**) Double color CK18/ZP staining shows intermediate filaments and ZP+ centrally located nucleus (arrowhead) and unstained adjacent structure resembling the polar body (arrow). **e**) Cell with centrally located ZP+ nucleus (arrowhead) and unstained fragmented adjacent structure (arrow). Note a lack of ZP+ intermediate filaments (compare with panel **a**) and surface ZP expression (solid arrow; compare with black arrow, panel **d**). **f**) Nuclear expression (arrowhead) of meiotically expressed PS1 carbohydrate ZP antigen. Note focally enhanced (coarse) staining (arrow) in one of the two rounded structures. **g**) Two cells showing a lack of nuclear PS1 expression (arrowheads), but strong staining is associated with adjacent polar-like bodies (arrows).

**Figure 4:**
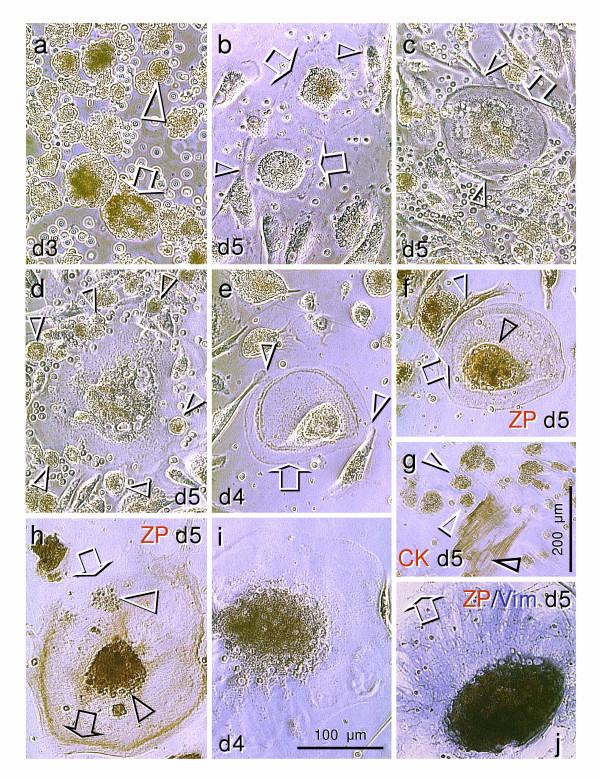
**Development of oocytes in day 3 to 5 mixed ovarian cultures. **Undifferentiated (stem) cells persist in all day 3 cultures (**a**). Under PhR+ conditions (**b-h**), the 100 μm oocyte-like cells were found on day 5 (arrows, **b**) with developing zona pellucida layer (arrow, **c**) in the presence of associated fibroblasts (arrowheads). Association of granulosa type cells (arrowheads, **d**) appears to stimulate advanced oocyte growth. Thick zona pellucida layer is apparent in some oocytes accompanied by fibroblasts on day 4 (**e**), and staining for ZP proteins on day 5 shows no change in the oocyte size but a lack of surface ZP expression (arrow, **f**). Staining for CK5,6,8,17 (**g**) shows cytoplasmic expression in mesenchymal type cells (black arrowhead) and non-specific staining (peroxidase expression) in the nuclei of other cells (arrowheads). **h**) Isolated large (200 μm) cells showed characteristics of secondary oocytes, including poor separation between the nucleus and cytoplasm (black arrowhead) expulsion of polar body (white arrowhead) and surface expression of ZP proteins (black arrow). The latter was, however, not apparent in the surface segment close to the polar body (white arrow). Under PhR- conditions, rare giant oocytes (300 μm) were found on day 4 (**i**). They lacked the germinal vesicle breakdown and surface expression of ZP proteins on day 5 (arrow, **j**). Bar in (**i**) for **a-f **and **h-j**.

**Figure 5:**
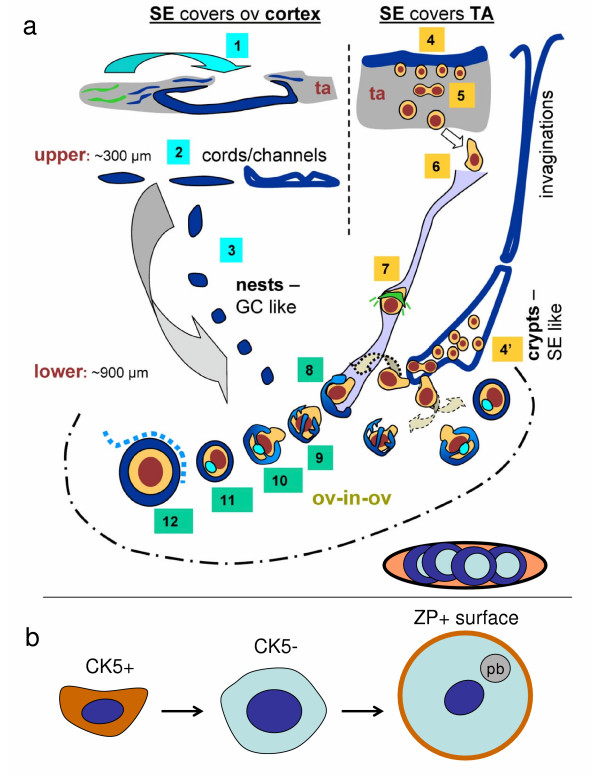
**Comparison of human oogenesis *in vivo *and *in vitro *a**) A working model of possible pathways for oogenesis and formation of primary follicles in adult human ovaries *in vivo *(adjusted from Ref. [6]). (1) Ovarian tunica albuginea (ta) stem cells (green color) first differentiate into the CK+ fibroblasts (blue color) and by mesenchymal-epithelial transition give rise to the SE cells directly covering the ovarian cortex (arched arrow). (2) Closing of TA results in the formation of epithelial cords in the upper ovarian cortex. (3) Fragmented epithelial cords give rise to the epithelial nests, which resemble primitive granulosa cells and descend into the lower ovarian cortex. (4) Depending on certain in situ (stromal and neuronal) and systemic (hormonal) influences, the TA progenitors differentiate into the SE cells covering TA, which may, by asymmetric division, give rise to the ZP+ germ cells. (5) These primitive germ cells symmetrically divide, descend into the ovarian cortex, and associate with adjacent cortical vessels (6). Intravascular transport (7) is associated with a substantial increase of germ cell size and with development of ZP+ anchors (green lines), which may serve to slow down the transport speed and signal the epithelial nests to associate with a particular vascular segment. 8) The intravascular germ cells differentiating into the oocytes are picked up by epithelial nests associated with the proper cortical vessels. Such oocyte-nest complexes show an "octopus-like" (9) formations during the early stage of assembly, and a formation of the Balbiani body during the intermediate stage (light blue body, 10). The Balbiani body persists in resting primary follicles (11), but diminishes upon the growth promoting signals, including Thy-1 dp signaling derived from the follicle-accompanying vessels (12, dashed line). An alternative pathway for the germ cell origin from TA precursors (4') consists of a constitution of cortical crypts formed by SE-like embryonal type cells, possibly originating from, but not necessarily connected with the deep SE invaginations, as evidenced from serial sections. The "alternative" pathway of germ cell origin may supply the oocytes directly to the neighboring nests (dashed arched arrows) and, via vascular transport (dotted arched arrow), saturate distant nests to form the primary follicles. The oocytes not utilized for follicular renewal accumulate in medullary vessels and degenerate (bottom right) [6]. **b**) *In vitro*, the CK5+ oocyte precursors may differentiate into CK5- cells (see Fig 2g), enlarge and undergo germinal vesicle breakdown followed by first meiotic division with a release of the polar body (pb) and ZP surface expression (brown color, see Fig. 3e and 4h).

In order to prevent loss of the OSE cells, no attempts were made to clean the ovarian surface from blood contamination accompanying the surgery prior to their collection. Consequently, the marked contamination with erythrocytes, along with our inability to distinguish cell types at this time, hindered our ability to count cells until the medium was changes after 24 hours of culture. The surface of intact ovaries was first gently scraped in the aseptic laminar flow hood with a sterile stainless steel surgery knife blade No. 21 (Becton Dickinson, AcuteCare, Franklin Lakes, NJ), leaving the blade edge behind the knife pass (pertains to data in Figs 1,2,3). This procedure was selected with an intent to include OSE and some adjacent TA mesenchymal cells. In some instances, a second type of culture was also established, where the cells collected from the ovarian surface were enriched with cells spontaneously released (without scraping) from dissected ovaries (pertains to data in Fig. 4). The intent of this approach was to test the activity of the alternative pathway for the origin of germ cells from cortical epithelial crypts (see Fig. 5a and Ref. [6] for details). Such cells were passed through a sterile 70 μm nylon cell strainer (BD Biosciences, Bedford, MA) to prevent contamination with larger type cells and structures.

The cells were collected into sterile petri dishes containing tissue culture medium supplemented with heat inactivated 20% fetal bovine serum (FBS; Gibco/BRL, Grand Island, NY) and antibiotics (50 μg/ml gentamycin, 100 U/ml penicillin, and 100 μg/ml streptomycin). The tissue culture media utilized was either Dulbecco's Modified Eagle Medium/Ham's F12, phenol red free (DMEM/F12; without estrogenic stimuli) or Dulbecco's Modified Eagle's Medium containing 25 mM HEPES, 4500 mg/L glucose, and phenol red (DMEM-HG; with estrogenic stimuli). The possible influence of additional steroid hormones or promoters of steroidogenesis present in the serum can not be ruled out. However, at the same serum concentration, there were apparent differences in the evolution and maturation of oocytes cultured in media with and without PhR (Fig. 4h–j). There was no other treatment imposed during the culture.

The cells were spun down (1000 × g, 5 min, 24°C), diluted in 0.75 – 1.5 ml of supplemented media, seeded in either 3 or 6 wells of a 24-well plate (250 μl per well) (Fisher Scientific, Pittsburgh, PA), and cultured in an humidified atmosphere with 5% CO_2 _at 37°C. The number of wells was chosen by the size of ovaries. Cells collected from small ovaries were seeded into 3 wells, and from larger ovaries into six wells. All ovaries involved in the experiment were anovulatory, as no corpora lutea were detected. The culture medium was changed once after 24 hours. This left only adherent (viable) cells in culture, and eliminated non-adherent cells and the majority of contaminating erythrocytes. The cell cultures were monitored daily by phase contrast microscopy and live cells evaluated by immunohistochemistry after 5–6 days from the initial seeding. Viability of cells was apparent from their active movement, changes in shape, and movement of their nuclei (compare Fig. 4e and 4f). The number of adherent cells in a single well of 24-well plate ranged between ~100 to 1000 during the late culture period (day 5 or 6).

### Single and double color peroxidase immunohistochemistry

The medium was aspirated from wells of the 24-well plate, and cells were allowed to dry under a fan before placing them in a ventilated hood overnight. This is a procedure we have utilized for cryostat sections. The bottoms of the wells were dried in the upright position and found macroscopically dry within a few seconds. The cells were fixed with 96% ethanol for 5 minutes, allowed to dry again, and incubated overnight (4°C) with primary antibodies against ZP proteins: rabbit-anti heat solubilized porcine zona [19] (HSPZ, 1: 20) or mouse monoclonal PS1 antibody recognizing meiotically expressed ZP carbohydrate antigen [20,22] diluted 1:10 in phosphate buffered solution (PBS), pH 7.22. The HSPZ and PS1 antibodies were originally donated by Dr. Dunbar, and an additional HSPZ antibody by Dr. Prasad. We also used mouse-anti human CK18, clone CY90, Sigma (diluted 1:50), mouse-anti human CK5,6,8,17, clone MNF116, DAKO Corporation, Carpinteria, CA (diluted 1:50), and mouse anti-human vimentin, clone V9, DAKO Corporation (diluted 1:50).

As a control, the HLA-DR antibody donated by Drs. Hilgert and Horejsi (tissue culture supernatant diluted 1:5), which does not react with OSE cells, was used. We used an HLA-DR antibody not reacting with fibroblast, granulosa, epithelial, germ or oocyte cell types, in order to identify activated tissue macrophages, but did not find any such cells in day 5 or 6 cultures. This does not exclude the possibility that activated tissue macrophages may have been present during the initial stages of culture.

After several washes in PBS (room temperature), the cells were incubated with peroxidase labeled corresponding secondary antibodies – goat anti-rabbit IgG, pre-absorbed with human serum (Jackson Immunoresearch, West Grove, PA), diluted 1:50; or swine anti-mouse IgG (donated by Dr. Peknicova), diluted 1:50 and absorbed with rat kidney homogenate to remove background as described previously [6]. After additional washes in PBS (room temperature), the bound antibodies were visualized by diaminobenzidine substrate as described previously [6], but without hematoxylin counterstain, covered with PBS, and images captured as described below. The cells stained for CK18 or ZP were processed further for dual color immunohistochemistry to identify co-expression of other proteins, and visualized with blue chromogen substrate as described previously [6]. Finally, the washed cells were covered with PBS containing 0.01% azide as a preservative.

### Image processing

Two independent observers evaluated the live cells by phase contrast and subsequent immunohistochemistry using an inverted microscope (NIKON, Nikon Inc., Instrument Division, Garden City, NY) equipped with a DEI-470 CCD Video Camera System (Optronics Engineering, Goleta, CA) with detail enhancement. The video images were captured by CG-7 color frame grabber (Scion Corporation, Frederick, MD) supported by Scion Image public software developed at the National Institutes of Health (Wayne Rasband, NIH, Bethesda, MD), and ported to Windows XP, 2002 release (Microsoft Corporation, Redmont, WA). To obtain figure panels, the captured video images were copied into Microsoft^® ^Power-Point^® ^97 SR-2 (Microsoft Corporation). Each image (including controls) was further copied into Microsoft Photo Editor 3.0 (Microsoft Corporation), and blue saturation adjusted (brightness 70, contrast 70, gamma 0.30). In total, more than 100 images were captured and stored.

## Results and discussion

### Oogenesis in vitro from ovarian surface epithelium (OSE) cells

We report data from pure OSE cultures of two cases investigated either under PhR- (age 47) or PhR+ medium conditions (age 40), and pure OSE and mixed cultures (OSE + stromal compartments; one patient) studied under both conditions (age 39 years). Cultures from the remaining two patients (ages 42 and 52 years) consisted of mesenchymal cells only.

#### Experiment 1 (Fig. 1)

Primary culture of cells scrapped from the surface of ovaries of the 47 year old female was maintained for 6 days in the Dulbecco's Modified Eagle Medium/Ham's F12 phenol red free (DMEM/F12), which was supplemented with 20% fetal bovine serum (FBS) and antibiotics. The 5–6 day interval was chosen since, except for fibroblasts, other cell types are not phenotypically distinguishable prior to day 4 (see Fig. 4). Phase contrast microscopy (Fig. 1a) shows differentiation of cells toward the mesenchymal (fibroblast) phenotype with perinuclear accumulation of cytoplasmic organelles (arrowhead).

Immunohistochemistry shows ZP expression associated with the nuclei and perinuclear space of mesenchymal type cells (arrowhead, Fig. 1b). The culture also showed clusters of cells of epithelial phenotype, which exhibited nuclear and perinuclear ZP expression (Fig. 1c). Occasionally, ZP+ cells of neural phenotype with extensions toward the mesenchymal cells (arrowhead, Fig. 1d) were observed, including growing cultures (inset). Fig. 1e shows mesenchymal type cells (arrowhead) and clusters (arrows) of small (15 μm) cells of granulosa cell phenotype. Beside their characteristic size, these granulosa type cells showed strong ZP expression, which has been found to be shared between oocytes and granulosa cells [6,23]. Furthermore, double color ZP/CK5,6,8,17 immunohistochemistry (Fig. 1f) showed additional dark blue staining (arrows) characteristic of granulosa but not germ or mesenchymal cells [6]. Note only a brown color in mesenchymal type cells (arrowhead). These observations indicate that numerous small cells exhibiting the phenotype and biochemical markers of granulosa cells can differentiate in the OSE culture.

An interesting question is why other cell types, in addition to the mesenchymal cells, evolved in this culture. As we have noted, the multipotential progenitor cells exhibiting a mesenchymal phenotype are capable of differentiation into distinct cell types, similar to stem cell cultures derived from other adult tissues [12-16]. In this culture, groups of cells exhibited either epithelial, neural, of granulosa cell phenotypes. We speculate that if a single mesenchymal cell in the given group decides to be, for example, an epithelial cell, the adjacent mesenchymal cells follow the same pattern. If no such decision is made, the cells in the group remain mesenchymal (compare groups of cells in Fig 1b–f). Hence, in this instance, the result is not an artifact of the medium used, but may be a case of distinct differentiation between groups of cells in the same culture.

#### Experiment 2 (Figs 2 and 3)

In contrast, cells from 40 year old female cultured in the Dulbecco's Modified Eagle's Medium containing 25 mM HEPES, 4500 mg/L glucose and phenol red (DMEM-HG; supplemented with 20% FBS and antibiotics as above) showed a uniform pattern of epithelial type cells, while no other cell types, including mesenchymal and granulosa cells, were present. Phenol red is a weak estrogen with apparent biological effects, and the estrogen receptor alpha binding affinity in Dulbecco's Modified Eagle's Medium is equivalent to levels of 17β estradiol as high as 0.45 nM [24]. This estrogenic binding affinity of phenol red is close to that of 17β estradiol serum level during the preovulatory peak [25]. High estrogen concentrations prior to ovulation are not only predictive of high oocyte quality [26], but they may be essential for stimulating oocytes to enter the first meiotic division and form large secondary oocytes. Figs 2a and 2b show phase contrast of large cells exhibiting an oocyte phenotype in the OSE culture maintained in DMEM-HG medium with phenol red for 5 days. The cell in Fig. 2b reached 180 μm in diameter and showed a centrally located 40 μm nucleus with nucleolus, and perinuclear accumulation of organelles (arrowhead).

Fig. 2c shows immunohistochemical staining of the cell in panel 2b for CK18. The nascent primary follicles in adult human ovaries show the gradual formation of CK18+ Balbiani bodies, which are undetectable by antibodies against other cytokeratins expressed by granulosa cells – CK5,6,8,17 [5,6,27]. A study by Cox and Spradling indicates that during Drosophila oogenesis, follicular cells are a source of mitochondria, which enter the oocyte cytoplasm via the "ring canal" to form the Balbiani body, thereby supplying virtually all of the mitochondria of the oocyte [28]. Dispersion of Balbiani bodies releases mitochondria required for the progressive growth of the oocyte. In a study of turkey hens, no Balbiani bodies were detected in stage I oocytes, but they appeared in stage II oocytes, and diminished in the oocytes of growing follicles, coinciding with the dispersion of mitochondria throughout the ooplasm [29]. Similar observations were reported in human oocytes [30]. The staining of the large oocyte in Fig. 2c shows diffuse CK18 immunoexpression with a preserved accumulation of staining around the cell nucleus (arrowhead). The perinuclear space also exhibits enhanced staining for ZP proteins shown by double color immunohistochemistry (CK brown/ZP blue color) in the same cell (arrowhead, Fig. 2d, compare with 2c). Control immunohistochemical staining is shown in Fig. 2e. Fig. 2f shows a cluster of OSE cells with perinuclear ZP expression (arrowhead), which differentiates into the large cells (arrows) that can be released into the free space. This resembles oocytes leaving the ovary during the perinatal period [31]. Fig 2g shows another cluster of OSE cells subjected to ZP/CK5,6,8,17 staining. Note the dark blue color in smaller OSE cells (arrowhead) and a diminution in larger cells (arrow).

We also observed large cells with two nuclei, where the ZP expression was apparent in the centrally located nucleus only (arrowhead vs. arrow, Fig. 3a; note ZP+ intermediate filaments). These cells of oocyte phenotype did not express CK5,6,8,17 [6] (Fig. 3b). The cell in Fig. 3c has two 60 μm nuclei and expresses the intermediate filament vimentin, a protein which plays an important role in the maturation and fertilization of eggs [21]. This observation indicates endoreplication of the oocyte nuclei in the absence of cell division, which has also been described in Drosophila eggs [32,33]. Interestingly, these multinucleated cells were not observed in the cultures without phenol red. The cell in Fig. 3d was stained for CK18 (brown color) and ZP proteins (blue). The centrally located nucleus again shows ZP expression (arrowhead), while the nucleus to the side, which resembles a polar body, was unstained (white arrow). Note a lack of surface staining (black arrow) vs. Fig 3e. The cell in Fig. 3e shows a ZP+ centrally located nucleus (arrowhead), with an adjacent unstained and fragmented structure (white arrow). Notably, there was surface expression of ZP proteins (solid arrow), which is important for the sperm-egg interaction [34]. Note the diminution of ZP+ intermediate filaments vs. Fig. 3a. Fig. 3f shows nuclear staining (arrowhead) for PS1, a meiotically expressed lactosaminoglycan-associated carbohydrate antigen of all three ZP glycoproteins [22,35], with more intense expression in the one of two nuclear segments, as indicated by the arrow. Two cells in Fig. 3g show the PS1 unstained nuclei (arrowheads) and adjacent PS1+ structures resembling expelled polar bodies (arrows). It has to be noted that similar couples of cells were often observed in PhR+ cultures, one of which was usually more developed (left cell in panel g) compared to the other (right).

### Oogenesis in "mixed" ovarian cultures

#### Experiment 3 (Fig. 4)

Next we decided to compare the PhR+ and PhR- conditions in pure OSE and mixed (OSE + stromal components) cultures derived from ovaries of a 39 year old female. It is important to note that all cells collected, including pure surface cultures, were passed through the cell strainer prior to processing and seeding, a procedure not applied in experiments 1 and 2. Utilization of a cell strainer ensured that mostly single cells, and not cell sheets (see Fig. 2f and 2g) were seeded. The mixed cultures were established to test the possible involvement of the alternative pathway for germ cell origin (Fig. 5a). Except for the occasional mesenchymal cells of the fibroblast phenotype, there was no evidence of cell commitment until day 4 of culture. However, the undifferentiated cells varied in the density of organelles and size, the latter ranging from 15 (arrowhead, Fig. 4a) to 50 μm in diameter (arrow).

On day 5 (end of the experiment) the cells collected from the ovarian surface did not progress beyond the state found on day 3, and no oocytes were detected regardless of the presence or absence of PhR. However, many cells of the oocyte phenotype were found in mixed cultures with PhR. They showed moderate (100 μm) size without zona pellucida (arrows, Fig. 4b), and were accompanied by fibroblasts (arrowheads). Larger oocytes (120 μm) accompanied by fibroblasts (arrowheads, Fig. 4c) showed a developing zona layer (arrow). On the other hand, an association of small round cells resembling granulosa cells (arrowheads, Fig. 4d) was characteristic for large oocytes (200 μm). The oocytes accompanied by fibroblasts (arrowheads, Fig. 4e) with thick zona pellucida (arrow) observed on day 4 showed no change in size on day 5 (Fig. 4f). Staining for ZP proteins showed a strong nuclear localization (black arrowhead, Fig. 4f) but no surface ZP expression (arrow). Note a well-defined separation between the nucleus and cytoplasm (a nuclear envelope, black arrowhead) of the oocyte. Also note the cellular and nuclear movements, when the Fig. 4e and 4f are compared. Fig. 4g shows cytoplasmic staining for CK 5,6,8,17 in mesenchymal cells (black arrowhead, note unstained nuclei) and moderate nonspecific staining (peroxidase expression) in the nuclei of other uncommitted cells (white arrowheads) in this culture. The nuclear peroxidase expression has been recently reported to increase in bovine oocytes and cumulus cells during *in vitro *maturation [36], and this may apply for many cells in this ovarian culture.

Some large cells in PhR+ mixed cultures showed characteristics of secondary oocytes with surface ZP expression (black arrow, Fig. 4h), expulsion of the polar body (white arrowhead) and poor nuclear/cytoplasmic separation (black arrowhead; see Ref. [37]). Interestingly, the surface ZP expression was absent in the oocyte segment toward where the polar body was extruded (white arrow).

Rare oocytes were detected in mixed cultures without PhR. The four day culture shows a giant (300 μm) oocyte with persisting germinal vesicle (Fig. 4i). Staining for ZP proteins (brown color) and vimentin (blue) on day 5 shows no surface ZP expression (arrow, Fig. 4j). This suggests that estrogenic stimuli may be required for the germinal vesicle breakdown and the development of secondary oocytes.

There are several issues to be addressed when experiments 1–3 are compared. For instance, experiment 3 provides evidence that ovarian fibroblasts may stimulate the oocytes to develop a thick zona pellucida layer, which allows their preservation until follicular growth is induced, when granulosa cells may contribute to oocyte enlargement. However, when compared to the experiment 2, no oocyte development was apparent in cultures derived from ovarian surface regardless of the presence of PhR. In experiment 3, however, the cells collected from the ovarian surface were passed through a cell strainer, which probably prevented the passage of the OSE cell sheets detected in the experiment 2 cultures (Fig. 2f and 2g). Apparently, cells derived from the OSE cell clusters are capable of transforming directly into secondary oocytes (Figs 2 and 3). On the other hand, experiment 3 has shown that an alternative origin of oocytes, e.g. from OSE cells in ovarian crypts (Fig. 5a), is possible in these mixed ovarian cultures in the presence of PhR. The likelihood that these mixed cultures are contaminated by migrating germ cells cannot be excluded, although very few oocytes were detected in PhR- mixed cultures. Nevertheless, many more oocytes of distinct sizes and stages of development were found in mixed cultures with PhR+. This suggests that estrogenic stimulation may play an important role in oocyte development. In addition, the age of the patients must be taken into account. Five day cultures of two patients (age 42 and 52) showed only mesenchymal cells. We suggested earlier that OSE in aging ovaries may be unable to produce germ cells, possibly due to the lack of local signals from activated tissue macrophages and neural cells, which may produce substances regulating the fate of progenitor (stem) cells [5] (see also Background).

## Conclusion

Altogether, our observations show for the first time that granulosa cells (Figs 1e and 1f) and oocytes (Figs 2 and 3) may develop directly from cultured OSE cells derived from adult human ovaries. This confirms our *in vivo *observations that in adult human ovaries, the OSE is a bipotent source of oocytes and granulosa cells. In addition, the oocytes developed *in vitro *undergo the first meiotic division, (Fig. 5b), after which they become suitable for fertilization. Oocytes may also develop in cultures containing ovarian stromal components. We speculate that such oocytes may originate from migrating germ cells or OSE invaginations (cortical crypts, Fig. 5a). The development and maturation of oocytes appear to be stimulated by estrogens. Depending on culture conditions (type of media utilized), processing of the collected cells, age of the ovaries, commitment of neighboring cells, and other local and hormonal factors, the progenitor mesenchymal cells in ovarian cultures may differentiate into additional cell types, including granulosa cells, or persist unchanged.

The ability to produce mature mammalian eggs from adult ovaries *in vitro *has several potential applications in the human and animal reproduction. Firstly, compared to the collection of follicular oocytes, the technique is easier (scrapping of the ovarian surface, with or without cortical component) and the yield might be higher for IVF and veterinary medicine purposes, since differentiation of primary oocytes *in vitro *may provide a larger number of secondary (mature) eggs. Secondly, for IVF purposes, this technique may be successful in women with premature ovarian failure, who lack follicles in their ovaries. Thirdly, the development and differentiation of oocytes from OSE precursors *in vitro *may help to better understand the process of oocyte renewal *in vivo*, and the role of accompanying granulosa and mesenchymal cells in the regulation of oocyte maturation or preservation. Fourthly, frozen OSE cells (oocyte stem cells) from younger females may be preserved for later production of fresh eggs. This may prevent the occurrence of fetal genetic alterations, which are often associated with pregnancies in advanced maternal age, possibly due to the lack of follicular renewal in aging ovaries. In addition, a colonization of premenopausal ovaries with younger oocyte and granulosa stem cells may establish a new cohort of primary follicles. This may result in a 10- to 12-year delay of the onset of natural menopause. Finally, the ovarian stem cells may serve as progenitor cells for several cell types for stem cell research, and fertilization of evolved mature human oocytes could result in the production of totipotent embryonic stem cells for research purposes and therapeutic applications.
